# Prospective Observational Study of Bisphosphonate-Related Osteonecrosis of the Jaw in Multiple Myeloma: Microbiota Profiling and Cytokine Expression

**DOI:** 10.3389/fonc.2021.704722

**Published:** 2021-06-24

**Authors:** Ashraf Z. Badros, Mariam Meddeb, Dianna Weikel, Sunita Philip, Todd Milliron, Rena Lapidus, Lisa Hester, Olga Goloubeva, Timothy F. Meiller, Emmanuel F. Mongodin

**Affiliations:** ^1^ Greenebaum Cancer Center University of Maryland School of Medicine, Baltimore, MD, United States; ^2^ University of Maryland Dental School, Baltimore, MD, United States; ^3^ Translational Laboratory Shared Services, University of Maryland School of Medicine, Baltimore, MD, United States; ^4^ Cytokine Core Laboratory, University of Maryland School of Medicine, Baltimore, MD, United States; ^5^ Institute for Genome Sciences, University of Maryland School of Medicine, Baltimore, MD, United States

**Keywords:** multiple myeloma, osteonecrosis of the jaw, bisphosphanates, microbiome, cytokines

## Abstract

**Purpose:**

Define incidence and risk factors of osteonecrosis of the jaw (ONJ) and explore oral microbial signatures and host immune response as reflected by cytokine changes in saliva and serum in multiple myeloma (MM) patients on bisphosphate (BP) therapy.

**Patients and Methods:**

A single center observational prospective study of MM patients (n = 110) on >2 years of BP, none had ONJ at enrollment. Patients were followed every 3 months for 18 months with clinical/dental examination and serial measurements of inflammatory cytokines, bone turnover markers, and angiogenic growth factors. Oral microbiota was characterized by sequencing of 16S rRNA gene from saliva.

**Results:**

Over the study period 14 patients (13%) developed BRONJ, at a median of 5.7 years (95% CI: 1.9–12.0) from MM diagnosis. Chronic periodontal disease was the main clinically observed risk factor. Oral microbial profiling revealed lower bacterial richness/diversity in BRONJ. *Streptococcus intermedius*, *S. mutans*, and *S. perioris* were abundant in controls; *S. sonstellatus* and *S anginosus* were prevalent in BRONJ. In the saliva, at baseline patients who developed BRONJ had higher levels of MIP-1β; TNF-α and IL-6 compared to those without BRONJ, cytokine profile consistent with M-1 macrophage activation. In the serum, patients with BRONJ have significantly lower levels of TGF beta and VEGF over the study period.

**Conclusion:**

Periodontal disease associated with low microbial diversity and predominance of invasive species with a proinflammatory cytokine profile leading to tissue damage and alteration of immunity seems to be the main culprit in pathogenesis of BRONJ.

## Highlights

All published data regarding BRONJ focused on patients who had active lesions. In this study we prospectively followed patients at high risk of BRONJ to define clinical factors and changes in immune cytokine milieu and oral microbiome before, during, and after developing of ONJ. Our findings support earlier reports that periodontal disease is a major risk factor for BRONJ; in this setting there was lower bacterial richness/diversity and predominance of invasive species and a cytokine signature consistent with proinflammatory profile, mostly noted in M-1 macrophage activation responsible for tissue damage and impaired healing. Our study supports dental evaluation before initiating BP therapy and aggressively treat periodontal disease. We suspect that changes in the oral microbiome diversity and richness will favorably alter the immune milieu ameliorating BRONJ.

## Introduction

Bisphosphonates (BP) are effective in the prevention and treatment of bone metastasis and hypercalcemia of malignancy. BP accumulates at sites of active bone formation and inhibits osteoclast function by altering cell differentiation inducing apoptosis, leading to reduction in bone resorption (1). After first report of bisphosphonate-related osteonecrosis of the jaw (BRONJ) in 2003, several case series confirmed this complication after long-term use of potent bisphosphonates and recently denosumab (2, 3). In multiple myeloma (MM) patients, the patient-year adjusted incidence of BRONJ is 1.1% during the first year of therapy, 4.1% from 2 to 5 years and increases up to 11% after 5 years of administration (4, 5). BRONJ is characterized by exposed necrotic bone in the oral-maxillofacial region that does not heal within 8 weeks after identification by a healthcare provider in patients receiving anti-resorptive agent and no prior radiation (6, 7). Clinically, patients are asymptomatic, few complain of pain and soft tissue swelling secondary to infection. The initiating event in the pathogenesis of BRONJ is usually loss of mucosal integrity from dental procedures or minor trauma that allows the formation of a microbial biofilm on the exposed bone leading to a chronic inflammatory cytokine response that further impairs normal healing and ultimately leading to bone necrosis (8).

In a prior retrospective case-controlled study of 22 MM patients who developed BRONJ, we identified the several risk factors including duration of BP therapy (median of 2 years), dental procedures, age (≥65 years), and long overall survival (9). Other studies described additional risk factors including diabetes, anemia, tobacco use, and periodontal disease (5, 10, 11). In a follow up study, we described the natural history of BRONJ among 97 MM patients who were observed prospectively for a median of 3.2 years following the diagnosis of BRONJ (12). The lesions healed in 62% of patients, resolved and recurred in 12% following re-initiation of BP, and did not heal in 26% of the patients. In this study, dental procedures preceded BRONJ in 47% of patients. Currently, there is no effective therapy for BRONJ, and the focus has been on prevention. Patients are advised to remedy any dental issues prior to starting BP and to avoid elective dental procedures while on therapy (13, 14). For established cases we recommend conservative management: stopping BP, and antimicrobial both systemically and as oral rinses with limited debridement (15). We carefully reinitiate bisphosphate therapy after complete healing of the ONJ lesions only in patients with relapsed MM and extensive bone disease.

Herein, we report our observational prospective study evaluating MM patients on long-term BP therapy (high-risk population for ONJ) to define the incidence, clinical risk factors and explore microbial signatures in the oral cavity and cytokine changes in saliva and serum in relationship to BP infusion and development of BRONJ.

## Methods

Patients were included in this observational study if they had confirmed MM diagnosis with active bone disease and had received bisphosphonate therapy for a minimum of 2 years. BP was administered as clinically. Patients should have no active BRONJ lesions at study entry; those with history of BRONJ were included if the lesions had completely healed for over 1 year. Patients should have an ECOG performance status ≤2 and a life expectancy greater than 24 months.

Patients provided written informed consent. The institutional review board at the University of Maryland approved the study. The study was conducted in accordance with the Helsinki and local laws. Patients may decide to withdraw from the study at any time. The study was funded by the NIH grant (R21 DE019509-01; PI AB). The Clinical Research Office at the Greenebaum Cancer Center, University of Maryland, performed data collection. The corresponding author (AB) wrote the first manuscript, all authors approved the manuscript and vouched for the accuracy and completeness of the data and analysis and adherence to the protocol.

Patients underwent dental and clinical medical assessments every 3 months (± 1 week) for 18 months. One oncologist (AB) and two dentists (TM, DW) performed the evaluation. Patients continued to receive standard of care; no changes were made in BP dosing and frequency. Patients received BP monthly for relapsed disease and every 3-month for those in remission and severe bone disease. Baseline dental evaluation included panoramic digital radiographs and documentation of pre-existing chronic conditions such as decayed, missing or fractured teeth and periodontal disease assessments including: extent (generalized *vs* localized), severity (destruction of soft and hard tissues), and activity (infection, teeth mobility, and/or pain).

Data collected included: baseline patients’ demographics and clinical characteristics, MM treatment, co-morbidities specifically diabetes mellitus and renal function, and smoking history. Dental data included: history of extractions, frequency of routine dental care (cleanings, restorations, and use of dental prosthesis). Patients who developed BRONJ data included time to onset from MM diagnosis, clinical features (stage, size, location, and severity), grade using American Association of Oral and Maxillofacial Surgeons’ staging guidelines.

### Correlative Studies

#### Sample Collection

Saliva was collected from each patient every 3 months (seven time points at months 0, 3, 6, 9, 12, 15, and 18), three samples were collected at each point; 5 min before BP infusion and 5 and 15 min after infusion. Whole unstimulated saliva (2–5 ml) was collected in sterile centrifuge tubes using standard techniques as outlined in the protocol. Three oral samples were collected from each patient at each visit: 5 min before BP infusion and 5 and 15 min after infusion.

Serum samples were collected at baseline and every 6 months before bisphosphate infusions (four time points; months 0, 6, 12, 18). Samples were maintained in an alcohol/ice bath and immediately centrifuged. Salivary supernatant and serum samples were divided in aliquots, de-identified, and frozen in sterile cryo-vials at −80°C until processed.

#### Microbiota Profiling

Microbiome profiling was performed on salivary samples collected after BP infusion every 3 months (seven time points) from 14 patients who developed BRONJ and compared with samples from patients who did not develop BRONJ (controls, n = 28). After missing samples and excluding samples with Good’s sequence coverage index <85%, 226 samples were analyzed using 16S rRNA gene sequencing.

DNA extraction: total metagenomic DNA was isolated using procedures in place at the University of Maryland School of Medicine’s Institute for Genome Sciences, as previously described. 16S rRNA gene sequencing: Microbiota profiling using 16S rRNA gene sequencing was performed as previously described. The assembled 16S sequences were then processed for taxonomic assignments. The cleaned amplicon sequences were clustered into Operational Taxonomic Units (OUT) using a 97% similarity cutoff based on Greengenes, Silva and Human Oral Microbial Databases (HOMD).

#### Cytokine Analysis

Cytokine expression was performed in the cytokine core laboratory at the University of Maryland. Cytokines were quantified using Luminex™ technology milliplex MAP kits (EMD Millipore, MA, USA), thus allows simultaneous quantification of multiple analytes in a single sample. Luminex assays are performed in duplicate and include an appropriate control in each assay. Luminex Multianalyte System is calibrated and maintained by certified and trained Luminex professionals.

Cytokine analysis in the saliva was examined at the baseline and at end of study; two samples at each time point, pre and 15 min after zoledronic acid infusion. A linear model with main effects of the measurement group and the interaction of the two was first fit for each cytokine. If the interaction term is statistically significant, that indicates there exists a group difference in terms of the slopes. Otherwise if the interaction term is not statistically significant, a reduced model was fit without the interaction term (i.e., assuming same slopes for the groups). Then we compare the intercepts of the fitted regression lines for different groups.

Cytokine analysis in the serum analyzed was collected at baseline (n = 42), midpoint (n = 35, 6–12 months samples), and end of study (n = 33, 18 months). An average of three samples per patient were available for a total of 129 samples. Saliva was collected before and 15 min after BP infusion at baseline (n = 42) and end of study (n = 39), four samples per patient for a total of 172 samples. Cytokines studied included: inflammatory cytokines (Interleukin 1 beta, Interleukin 6, Interleukin 17, Tumor necrosis factor-alpha, Transforming growth factor beta 1, Macrophage Inflammatory Protein-1 alpha, Macrophage Inflammatory Protein-1 beta and Matrix metallopeptidase 9); bone turnover markers (Osteopontin, ostiocalcin, Receptor activator of nuclear factor kappa-B ligand, Osteoprotregerin); and angiogenesis growth factors (Vascular endothelial growth factor and epidermal growth factor).

#### Study Objectives and Statistics

The primary objective of the study was to estimate the incidence of BRONJ in MM patients after long-term BP use and significant risk factors. Exploratory correlative focusing on bacterial colonization and host cytokine responses were evaluated longitudinally during the 18-month study period.

Microbiota analysis: sequences were assembled from pair-end reads across all samples included in the final analysis. After filtering and chimera removal, sequences were assembled into Operational Taxonomic Units (OTUs). The median number of sequences were compared between BRONJ and controls. Taxonomic binning of 16S reads was performed based on three different databases: Greengenes, Silva, and HOMD. OTUs from Greengenes, Silva, and HOMD were compared. HOMD provided the highest taxonomic assignments at the species level (data not shown) and was therefore used for subsequent analyses. Microbiota diversity analysis was analyzed with the standard analysis tools implemented in Qiime, as well as with Phyloseq, an R package dedicated to the object-oriented representation and analysis of microbiome census data in R (www.r-project.org). Alpha-diversity (a measure of intra-sample diversity) and beta diversity (a measure of inter-sample diversity) were reported. Alpha diversity was assessed using the observed diversity (total number of OTUs, a measure of community richness), the Chao diversity, and the Shannon diversity indexes. Alpha diversity of the samples was measured by observed species, the Shannon diversity, and the Chao1 index. The observed species index measures the number of different species per sample which is defined as “richness.” The Chao1 index is also a qualitatively measure of alpha diversity which, beside species richness, takes into account the ratio of singletons (n = 1) to doubletons (n = 2) giving more weight to rare species. However, regarding diversity, not only the qualitative amount of species, but also the abundance of the species must be taken into account. The relative abundances of the different species making up the samples’ richness are defined as “evenness”. The Shannon-diversity index relates both, OTU richness and evenness. Beta-diversity was assessed based on the Jensen-Shannon divergence and Bray-Curtis dissimilarity measure. Determination of statistical significance for OTU relative abundance levels and comparison of diversity across samples or group of samples were performed using the DESeq2 function in R. Differences were considered statistically significant if their associated P value was lower than 0.05.

Cytokine levels are affected by inherent factors, by bisphosphonate infusion schedule, disease status but also by frequency of zoledronic acid infusions, the correlation with each was examined in our study population. Cytokine analysis in the serum; a linear mixed model fitted by the PROC MIXED procedure in SAS was used to test for each cytokine; the same null hypothesis that the mean cytokine levels are the same between different groups [groups included bisphosphonate schedule (1 month *vs* 3), BRONJ status (developed ONG or not), or MM status (remission or relapsed)]. A statistically significant test indicates that at least at one time point, there exists a difference in mean cytokine level.

## Results

The study included 110 MM patients. One hundred patients received BP >2-years. The median time from MM diagnosis to study entry was 3.7 years (range: 2.5–13); 10 patients were newly diagnosed (control) with a median of 7 months (range: 1–9) from MM diagnosis. Patients’ demographics at study entry are described in **Table 1**. Patients were receiving monthly zoledronic acid infusions (n = 75) or every 3 months (n = 35) if they were in remissions. Twenty-five (23%) patients had diabetes mellitus and 32 (29%) were daily smokers. MM was in remission in majority of patients (n = 87, 80%): complete remission (n = 35, 32%) and partial remission (n = 52, 47%). MM therapy included: lenalidomide maintenance (n = 59); 10 newly diagnosed patients were receiving induction with bortezomib, lenalidomide, and dexamethasone; while 24 patients were not receiving any MM therapy. While on study, 50 patients progressed and received salvage therapies that included: bortezomib (n = 14), lenalidomide (n = 6), carfilzomib (n = 16), or other clinical trials (n = 14). Thirteen patients had died during the study follow up: nine from complications of relapsed MM and four from other causes. Eighteen patients withdrew consent for samples collection (saliva and blood) and study dental evaluations but continued MM follow up.

**Table 1:** Demographics and characteristics of the study population.

	Overall	No BRONJ	Developed BRONJ
	N = 110 (100%)	N = 96 (88%)	N = 14 (12%)
**At Study Entry**			
Age (range, years)	57 (33–81)	57 (45–78)	60 (33–81)
Males	68 (62)	59 (61)	9 (64)
Race			
○ Caucasians	58 (53)	51 (53)	7 (50)
○ Blacks	49 (45)	43 (45)	6 (43)
○ Others	3 (2)	2 (2)	1 (1)
MM Dx to study, median (range)			
○ High risk (n = 100)	3.7 (2.5–13)		
○ Newly Dx (n = 10)	0.6 (0.1–0.8)		
MM Dx to BRONJ, median (range)			5.7 (1.9–12)
Isotype			
○ IgG, IgA, Free Light chain	62, 29, 19	56, 25, 14	6, 4, 4
Prior SCT	104	94	10
MM Response at study entry			
○ CR	35	31	5
○ PR	52	48	4
○ PD	23	18	5
MM Therapy at study entry			
○ Lenalidomide	59	53	6
○ Carfilzomib	16	11	5
○ Other	11	8	4
○ No therapy	24	24	–
Medical History			
○ Diabetes mellitus	25	21	4
○ Smoking; continued smoking	32; 6	27; 2	5; 4
Dental History			
○ Followed dental care	66	54	9
○ Periodontal Disease			
- Mild	45	39	6
- Severe	47	44	3
○ Decay and Fracture	15	15	–
○ Using Dentures	15	14	1
**While On Study**			
Zoledronic acid infusions			
○ 1 Month	75	66	9
○ 3 Months	35	30	5
MM Relapse	50	42	8
Dental Extractions	10	5	5
○ Healed (8 weeks)	8	5	3
○ Did not heal	2		2

BRONJ, bisphosphonate related osteonecrosis of the jaw; MM, multiple myeloma; Dx, diagnosis; CR, complete response; PR, partial response; PD, progressive disease.

Of the 110 patients, 92 completed the study; 66 patients continued routine dental procedures. The predominant dental findings at baseline included: decay/fractured teeth (n = 15); periodontal disease was very common and classified as mild (gingival inflammation, n = 45), moderate (>50% loss of attachment, n = 44), or severe (n = 3). Fifteen patients had dental prostheses. All 110 subjects reported at least one previous extraction prior to development of MM; 10 patients had dental extractions while on study. Eight healed normally and two went on to develop BRONJ. Baseline panoramic digital radiographs were available for 109 patients; 10% (11 of 109) had mandibular and/or maxillary lytic lesions consistent with myeloma bone disease.

Fourteen patients developed BRONJ; 12 during the 18 months of follow up. Two patients developed BRONJ at 19 and 20 months, they were included in this analysis, dental characteristics are provided **Table 2**. Two patients with previously documented BRONJ, which had healed over 1 year prior to study entry, both developed new lesions at other sites. Median time from MM diagnosis to BRONJ was 5.7 years (the 95% CI: 1.9–12.0). Nine patients were in remission with regards to MM and were receiving monthly zoledronic acid. Most BRONJ lesions occurred in the mandible. Nine developed spontaneous lesions (without any known invasive procedures or trauma). Panoramic images for BRONJ were non-specific revealing few cases of minor sclerotic changes and bony sequestrates; overall imaging was not helpful is staging or assessment of active lesions. BRONJ healed in nine patients at a median of 6 months, with recurrence noted in three patients. Over the follow up period, it is worth noting that there was no increase in stage or severity of any BRONJ lesions, probably reflecting our conservative, non-surgical, management.

**Table 2:** Characteristics and outcome of BRONJ patients.

	BRONJ Patients N = 14 (%)
**Predisposing factors**	
○ Spontaneous	9
○ Dental Extraction	5
**MM status at BRONJ onset**	
○ CR	5
○ PR	4
○ PD	5
**MM Therapy at BRONJ onset**	
○ Lenalidomide	8
○ Carfilzomib	3
○ Other	3
○ Zoledronic acid	
▪ Monthly	9
▪ 3 months	5
**Symptoms**	
○ Mild (exam finding)	10
○ Severe pain, swelling	4
**AAOMS staging**	
○ 1	1
○ 2	11
○ 3	2
**Location of the lesions**	
○ Mandible	11
○ Maxilla	1
○ Both	2
**BRONJ Outcome**	
○ Healed	9
○ Non-healing	5
○ Healed and recurred	3

BRONJ, bisphosphonate related osteonecrosis of the jaw; AAOMS, American Association of Oral and Maxillofacial Surgeons.

The main predisposing risk factor for BRONJ was preexisting chronic moderate/severe periodontal disease. Surprisingly, routine dental care did not clearly prevent development of BRONJ in our patient population. There was no association between BRONJ and history of diabetes, smoking status and no relationship to MM disease status (remission *versus* progression). Less frequent infusions of BP every 3 months did not decrease the risk of BRONJ, although the numbers were small.

### Correlative Studies

#### Microbiome Profiles

**Figure 1:**
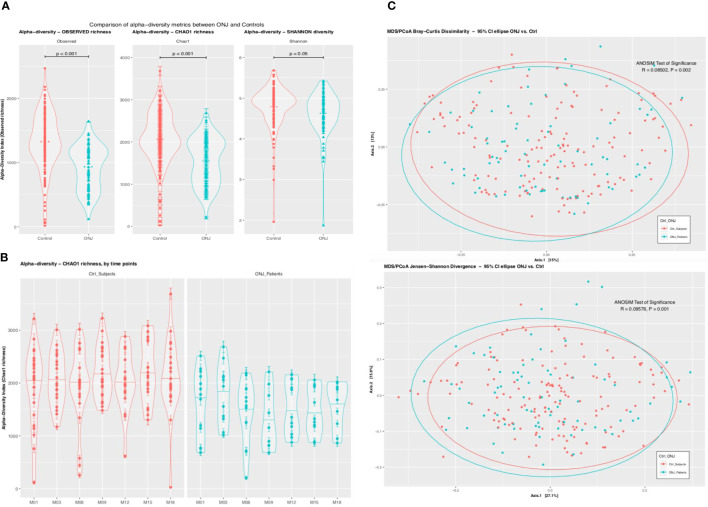
**(A)** Comparison of Alpha Diversity Metrics Between BRONJ and Control. Violin plots comparing the alpha-diversity metrics for richness (Observed and Chao1) and diversity (Shannon) between control (red) and BRONJ (blue). The graphs show significantly lower richness and diversity in BRONJ patients. The significance of difference was calculated using Kruskal-Wallis test with Conferroni correction. **(B)** Comparison of Alpha Diversity (Chao1 Richness) Between BRONJ and Control across study time points. **(C)** Principal coordinate analysis (PCoA) of oral cavity microbiota using Bray–Curtis dissimilarity matrix (top) and Jensen Shannon Divergence (bottom). Two-dimensional PCoA was used to describe the relative abundance of oral microbiota. Each point represents a single sample and is colored red for controls and blue for BRONJ.

Analysis of alpha-diversity metrics of showed a significantly lower bacterial richness (Chao1 index) and diversity (Shannon index) in BRONJ patients compared to controls (P < 0.001 for richness and P < 0.05 for diversity) (**Figure 1A**) (16). When analyzing the Chao-1 richness for each individual time points, we did not find any statistically significant difference in diversity between BRONJ and control subjects at study entry; however significant differences in diversity were observed starting at 6 months, suggesting predominance of specific species in BRONJ patients, even before development of BRONJ (**Figure 1C**). Similarly, bacterial community structure comparisons using beta-diversity analyses of Bray-Curtis dissimilarity showed that the oral microbiota in BRONJ subjects was significantly different to that of control patients (R = 0.085, P < 0.002) and the Jensen-Shannon divergence (ANOSIM test of significance R = 0.096, P < 0.001) (**Figure 1B**) (17).

**Figure 2:**
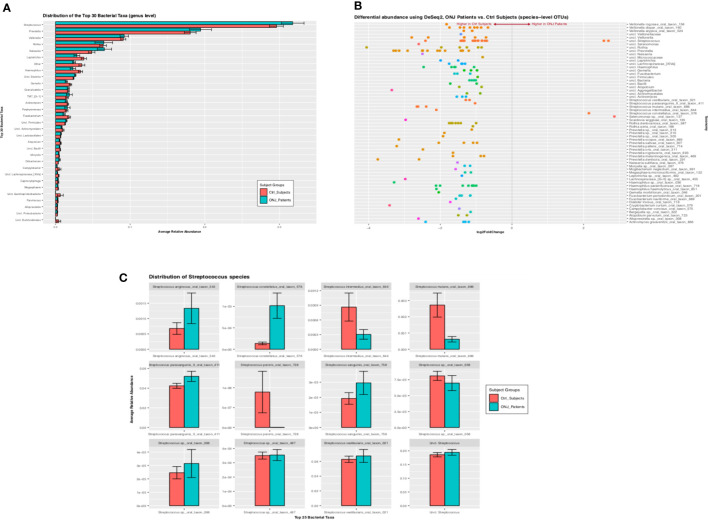
**(A)** Bar graph showing the distribution of the 30 top bacterial taxa (assigned at the genus level) between BRONJ and controls. Relative abundance of three genera (*Streptococcus*, *Prevotella*, and *Veillonela*) is seen in both BRONJ and controls. **(B)** Differential Relative Abundance Analysis Using Deseq2. Pairwise comparison of the relative abundance of Operational Taxonomic Units (OTU) at species level; positive values of the Log2 fold change indicate higher relative abundance in BRONJ and negative values indicate higher relative abundance in control. **(C)** Distribution of Streptococcus species between Control (red) and BRONJ (blue).

Among 6,313 OTUs, 5,617 (89%) were classified at the phylum level, 5,185 (82%) at the class level, 5,065 (80%) at the order level, 4,755 (75%) at the family level, 4,546 (72%) at the genus level, and 2,150 (34%) were classified at the species level. The top 30 genus- level OTUs that were significant when controlling the false discovery rate at 0.05 are shown in **Figure 2A**. *Streptococcus*, *Prevotella*, and *Veillonella* species were the most abundant taxa both in control and BRONJ patients, followed by *Rothia*, *Neisseria*, *Leptotrichia*, *Haemophilus*, *Gemella*, *Granulicatella*, *Saccharibacteria* (TM7), *Actinomyces*, *Porphyromonas*, and *Fusobacterium* species. All taxa were more abundant in control compared to BRONJ patients, with the notable exceptions of Streptococci and Selenomonas sp (**Figure 2B**). We further evaluated the distribution of Streptococcus species in our patient cohort: *Strep. intermedius*, *S. mutans*, and *S. perioris* were significantly more abundant in control patients, *S. constellatus* and *S. anginosus* were more prevalent in BRONJ patients even before developing BRONJ (**Figure 2C**).

#### Cytokine Profile

In the saliva, at baseline and in pre-zoledronic acid infusions, patients who developed BRONJ had higher levels of MIP-1B [14 (SD: 30) *vs* 6 (SD: 2), p = 0.01]; OPG [213 (SD: 39) *vs* 125 (SD: 26), p = 0.07]; TNF [10 (SD: 3) *vs* 3 (SD: 2), p = 0.09], and IL-6 [15 (SD: 4) *vs* 3 (SD: 3), p = 0.02] compared to those without BRONJ. Interestingly, at 18 months, patients on zoledronic acid monthly *vs* every 3 months had lower levels of MIP-1 A [6 (SD: 4) *vs* 20 (SD: 5), p = 0.03]; higher levels of MMP [60,784 (SD: 11,359) *vs* 107,106 (SD: 12,294), p = 0.01] and lower RANKL/OPG ratio at [0.3 (SD: 0.1) *vs* 0.5 (SD: 0.1), p = 0.01]. Patients in CR had lower levels of EGF [616 (SD: 577) *vs* 2,625 (SD: 548), p = 0.02] and higher OPG levels [139 (SD: 28) *vs* 72 (SD: 24), 0.03] compared to patients in PR.

In the serum, patients with BRONJ have significantly lower levels of TGF beta (0.006) and VEGF (p = 0.03) over the study period. Interestingly, those receiving monthly zoledronic acid had higher levels of RANK-l (p = 0.04) as expected physiologically.

## Discussion

BRONJ is a complicated process that involves interplay of drug effect on bone and/or oral mucosa, oral microbiome-host interactions, and alteration of immune and inflammatory cytokines. In this prospective evaluation of BRONJ in high-risk MM population, the incidence of BRONJ was 13% after prolonged exposure to zoledronic acid. Thus supports the current practice to stop therapy after 2 years; for those with extensive bone disease continuation every 3 months seems reasonable although efficacy and risk of BRONJ requires further study. The rigorous in this follow up study identify 9 of 14 patients who developed BRONJ spontaneously with no symptoms, suggesting that the role of extraction as an initiating event is limited and many cases are missed in routine practice. All BRONJ patients had periodontal disease; which is the main risk for osteonecrosis identified in our study. This level of periodontal disease is expected in the age group of this study population (18, 19).

The microbiome profile of MM patients who developed BRONJ showed lower diversity, higher relative abundance of oral pathogens, and a lower commensal bacterium—compared with patients who did not develop BRONJ—indicating that the oral microbiota play an important role in initiating and/or maintaining BRONJ (20–24). Other studies have shown lower microbiome diversity and higher abundance of Streptococci in the exposed bone of patients with BRONJ (22, 25). Our study provides prospective unbiased data, allowing the characterization of the entire salivary microbiome in MM patients months prior to the development of BRONJ lesions. Our focus was on the microbial environment predisposing to BRONJ rather than the microbiome colonizing the site of active lesions, previously reported. Using an established “Human Oral Microbiome Database-HOMD”, (26) we identified 72 and 34% of taxa to the genus and species level, respectively, in our patients. Interestingly, true pathogens that can cause suppurative and invasive infections such as *Strep. constellatus* and *Strep. anginosus*, *Strep. anginosus* group, were more predominant in patients with BRONJ before developing lesions, suggesting a possible causative/promoting effect (6, 25, 27). Conversely, *Actinomyces* sp., *Fusobacterium nucleatum*, and *Eikenella* sp. which were previously reported as abundant at the site of bone necrosis were not found to be significant in the oral microbiome of our cohort of patients (28). This might reflect a late colonization of exposed bone, rather than an early causative event. Our results suggest that the initiation and progression of BRONJ is associated with diminished bacterial diversity and abundance of specific pathogens in a susceptible host.

Although many studies suggested that prolonged suppression of osteoclasts—the main biological effect of BP—is the culprit in BRONJ pathogenies. In the current study, bone turnover markers in the serum and saliva had no correlation with BRONJ. Interestingly, the cytokine signature in the saliva of BRONJ, high levels of MIP, IL-6, and TNF, are consistent with an inflammatory response of type-1 macrophages (M-1, pro-inflammatory) (29). It is interesting that macrophages are remarkably similar to osteoclasts and exposure to BP affects their phenotype and ability to activate and respond to infections (30). These M-1 responses can lead to secretion of nitric oxide and reactive oxygen species degrading the extracellular matrix resulting in sustained mucosal damage and death of the underlying bone (31). In normal physiologic conditions, M-1 response is followed by type-2 macrophages response, which are responsible for regenerative healing, this appears to be blunted in BRONJ patients (32). This is particularly interesting as blockade of the RANK–RANKL interaction by denosumab, another drug with high risk of BRONJ, could affect monocyte migration and modify macrophage functions, more M-1 phenotype, similar to that created by bisphosphonates (33). Last, serum VEGF is a potent angiogenesis regulator with effects on endothelial cells. Lower levels noted in BRONJ patients lead to impaired endothelial proliferation/migration and repair of mucosal lesions (34–36). This is further augmented by low levels of TGFβ-1 adding to endothelial dysfunction and inhibition of wound healing (37). An attempt to link each of the cytokine measurements to overall variation of oral microbiota was not useful due to low number of samples and dominance of few species in BRONJ patients.

In conclusion, this is the first, to our knowledge, observational prospective study to evaluate MM patients on long-term BP therapy exploring multiple time points for microbial signatures and cytokine changes before and after development of BRONJ. The major risk factor for BRONJ was periodontal disease. ONJ patients had lower microbial diversity and predominance of invasive species such as *Strep. constellatus* and *anginosus*. Cytokine changes in ONJ patients were consistent with proinflammatory profile of M-1 macrophage activation, responsible for tissue damage. These findings provide insight into the oral microbiome-host interactions, and alteration of immune and inflammatory cytokines response as a culprit in pathogenesis on BRONJ.

## Data Availability Statement

The raw data supporting the conclusions of this article is available on request. Requests to access the datasets should be directed to the corresponding author.

## Ethics Statement

The studies involving human participants were reviewed and approved by University of Maryland IRB. The patients/participants provided their written informed consent to participate in this study.

## Author Contributions

AB, designed the study, obtained funding-NIH grant (R21 DE019509-01), identified the patients, collected the data, and written the first draft of the paper. MM and EM performed and analyzed the microbiome data. DW and TFM performed all the dental evaluation and provides the dental data. SP, TM, and RL supervised the research and collected and prepared the blood and saliva samples. LH and JH performed the cytokine analysis. OG performed the statistical analysis for the cytokine data. All authors contributed to the article and approved the submitted version.

## Funding

NIH grant R21 DE019509-01 (PI: AB).

## Conflict of Interest

The authors declare that the research was conducted in the absence of any commercial or financial relationships that could be construed as a potential conflict of interest.
